# Intergenerational Conflict Among Asian Immigrant Families and Psychological Well-Being: Results from PIETY Study

**DOI:** 10.1093/geroni/igab046.2907

**Published:** 2021-12-17

**Authors:** Fatima Abdi, Stephanie Bergren, Lisa Lanza, XinQi Dong

**Affiliations:** 1 Rutgers University, New Brunswick, New Jersey, United States; 2 Rutgers University, Rutgers Institute for Health, New Jersey, United States

## Abstract

Research suggests that stress from migration and cultural adjustment may lead to intergenerational conflict (IC) within Asian immigrant families. Current research reports management of IC but fails to acknowledge the consequences it may have on offspring. The PIETY study, a longitudinal study of Chinese adult children (n = 547) in the greater Chicago area, aims to examine the relationship between IC and psychological wellbeing in children of Asian immigrant families. IC is assessed by the sum of items on conflicting opinions with parents based on finances, health, parenting, and lifestyle. Psychological wellbeing was measured by the Perceived Stress Scale with a cutoff value greater than or equal to 14, R-UCLA Loneliness Instrument scored on a binary scale, and Hospital Anxiety and Depression Scale (HADS) Anxiety Subscale with a cutoff value greater than or equal to 8. Logistic regression was conducted and controlled for age, gender, education, income, marital status, and household composition. Every one-point higher conflict with parents was associated with being 2.31 times more likely to experience stress for the adult child (OR: 2.31, 95% CI: 1.49-3.57, p<.001) and being 4.56 times more likely to experience loneliness (OR: 4.56, 95% CI: 2.79-7.43, p<.001). IC, however, had a nonsignificant positive association with anxiety in adult children. The association between IC and psychological wellbeing suggests that conflict is a result of complex factors, for which interventions could be developed to improve psychological wellbeing and resiliency in families who continue to navigate cultural changes in a foreign land.

